# Hoffman’s Exercise for Breastfeeding Support Among Postnatal Mothers With Nipple Defects: A Scoping Review and Exploratory Meta‐Analysis

**DOI:** 10.1155/tbj/2162565

**Published:** 2026-02-02

**Authors:** Hester Lacey, Nityanand Jain, Ian C. C. King

**Affiliations:** ^1^ Flinders University, Sturt Road, Bedford Park, 5042, South Australia, Australia, flinders.edu.au; ^2^ University Hospitals Sussex NHS Foundation Trust, Eastern Road, Brighton, BN2 5BE, UK; ^3^ Independent Statistical Consultant, Chandigarh, 160036, India; ^4^ Queen Victoria Hospital NHS Foundation Trust, East Grinstead, RH19 3DZ, UK, qvh.nhs.uk

**Keywords:** breastfeeding, effectiveness, flat nipple, Hoffman’s exercise, nipple inversion

## Abstract

**Background:**

Hoffman’s exercise is a widely promoted nonsurgical technique to assist breastfeeding among postpartum mothers with inverted or flat nipples. Prior reviews have suggested benefit but did not account for differences in effect by comparator, nor did they distinguish between‐group from within‐group change. This limits clinical guidance and planning for rigorous trials.

**Aim:**

To present an analytic discussion that is comparator‐aware, separates effect types, and foregrounds uncertainty; clarifying what the literature can credibly support and highlighting gaps in study design and reporting standards.

**Discussion:**

Across a small and heterogeneous evidence base (*n* = 10 studies), the quality of primary studies was critically low. This limited the appropriateness of definitive synthesis using meta‐analytic methods. Nonetheless, acknowledging the risk of estimate inflation, we still conducted an exploratory, hypothesis‐generating analysis. Stratification by comparator suggested a possible benefit versus routine care, but no clear advantage against the inverted syringe technique. Within‐group pre–post improvements were common yet remained noncausal and vulnerable to confounding. Coupled with our wide prediction intervals and high risk of bias, these findings suggest that confident claims of effectiveness maybe premature in this population.

**Conclusions and Recommendations:**

Current evidence neither supports strong effectiveness claims for Hoffman’s exercise nor warrants abandoning the technique outright. Clinically, we suggest offering the exercise as part of a broader lactation‐support bundle rather than presenting it as a stand‐alone, proven or validated intervention. More robust data are needed to determine the clinical effectiveness of the technique.

## 1. Introduction

Nipple inversion is described as the projection of the nipple posterior to the areolar plane. Flat or inverted nipples are relatively common, affecting an estimated 10%–20% of the population [1]. The condition is usually congenital and less often acquired. It is frequently bilateral with many individuals remaining asymptomatic until breastfeeding [1]. Apart from the cosmetic concerns, nipple inversion can impede breastfeeding through suboptimal latch and positioning, cause maternal nipple pain and lactation‐related infections, which may contribute to early cessation of breastfeeding in the postnatal period [2]. Accordingly, the severity of nipple inversion has been classified into three grades that reflect the degree of fibrous tethering and ductal distortion [3]. These grades are often reported to correlate with the ease of manual eversion and the likelihood of treatment success. Higher grades are associated with greater aesthetic concern and a higher risk of functional impairment [3].

Current management options span non‐surgical techniques and surgical interventions. Non‐surgical approaches are typically first‐line for Grade 1 and 2 inversions, reserving surgery for more severe cases or when conservative measures fail [4]. One such non‐surgical approach is Hoffman’s exercise. First described in 1952, the manoeuvre is generally described as safe and painless [5]. Although the exercise involves supervised instruction in the initial stages, it can be performed independently once the patient is proficient. The exercise involves repetitive, circum‐areolar downward traction to gradually evert the nipple [6]. It is posited to work by stretching the fibrous adhesions at the nipple base, thereby improving protrusion, increasing tissue pliability and enhancing local tissue perfusion [7].

## 2. Current Evidence

In recent years, a few reviews have summarised the literature on the effectiveness and utility of Hoffman’s exercise and other interventions in antepartum and postpartum women with nipple defects [4, 6, 8]. Most concluded that Hoffman’s exercise is an effective nonsurgical option, reporting improvements in nipple aesthetics and breastfeeding success. However, in our opinion, the certainty of evidence to support these conclusions remains limited. We were particularly concerned that most primary studies relied on quasi‐experimental designs with a high risk of bias. The situation is compounded by the heterogeneity in technique administration, comparators, considered end‐timepoints, and outcome measures.

In fact, newer data from grey literature seems to suggest no clear advantage of the technique for either aesthetic or functional endpoints. While some of this inconsistency may reflect differences in assessment tools and the inherit subjectiveness in these assessments, we argue that claims of effectiveness may have been premature, especially in the absence of adequately controlled, comparator‐specific trials. Against this backdrop, we decided to scope the literature to present an evidence‐informed discussion rather than a definitive systematic review or meta‐analysis. We also reassessed between‐group differences and within‐group pre–post changes, alongside performing a risk‐of‐bias appraisal.

Our goal was not to over‐interpret or inflate imprecise estimates but to clarify what current evidence can quantitatively and credibly support and, equally important, what it cannot. We use our findings to outline critical methodological priorities for future studies and pragmatic considerations for clinical practice. By framing the field’s evidential gaps and offering a roadmap for rigorous studies, this discussion aims to stir and move the conversation from enthusiasm and reliance on anecdotal wisdom to the need for decision‐ready evidence for clinicians, patients, and policymakers.

## 3. Methods

As we conducted this as an exploratory and theoretical analysis to advance further discussion in the field, the review was considered scoping in nature and did not require pre‐registration of the protocol. The PICO (patient, intervention, control, outcome) scheme was applied to formulate the primary and secondary research question: (P) Adult pregnant patients diagnosed with flat or inverted nipples, irrespective of their stage of pregnancy; (I) Hoffman’s exercise with a clear description of technique and frequency of administration; (C) any non‐surgical intervention including routine care; and (O) improvement in breastfeeding uptake as measured using any validated questionnaires.

**FIGURE 1:**
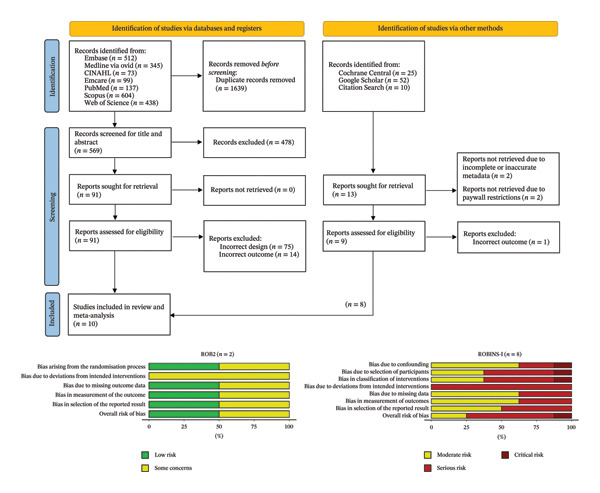
PRISMA flowchart for study screening and selection. Risk of bias analysis is presented based on study design—ROB2 for randomised controlled trials; ROBINS‐I for interventional non‐randomised studies.

The primary research question so formulated was the potential efficacy of Hoffman’s exercise over routine care and/or other non‐surgical interventions. The secondary research question was to assess the within Hoffman’s exercise group, pre–post improvement to characterise the direction and magnitude of change after Hoffman’s exercise, to assess the questionnaire responsiveness and to obtain design parameters for informing future trials. We systematically searched seven biomedical databases (CINAHL, Embase, Emcare, MedLine, PubMed, Scopus, and Web of Science) along with scoping grey literature sources and performing a bi‐directional citation searching (Figure 1). A basic search string comprising of terms such as ‘Hoffman’s exercise’, ‘breastfeeding’, ‘nipple inversion’ and ‘flat nipple’ was adopted. The search was performed independently by HL and NJ for every database, and results were exported to Covidence. Automated removal of duplicates was done using the Covidence software.

The inclusion criteria followed the PICO criteria with no restriction on publication date or language of publication. We excluded conference abstracts, posters, letters and editorials if they did not report the required metrices. Animal studies were not considered. Reviews were also excluded. Title and abstract screening were done by HL and NJ independently, with conflicts resolved by mutual discussion with ICCK (Consultant in Plastic, Reconstructive and Aesthetic Surgeon). A 95% agreement was observed between NJ and HL. Full‐text PDFs of potentially eligible studies were obtained from publisher’s website or institutional repositories. They were assessed for inclusion by NJ and HL independently with a 100% concordance.

Accordingly, we identified 10 studies that described the effectiveness of Hoffman’s exercise in antepartum and postpartum women with nipple defects (Table 1) [7, 9–17]. We selected these 10 studies since they quantitatively (*though prone to subjectiveness*) assessed the impact of Hoffman’s exercise on breastfeeding uptake using validated questionnaires such as LATCH, Bristol Breastfeeding Assessment Tool (BBAT) and Modified Via Christi Breastfeeding Assessment Scale (MVCBAS) [18–20]. Briefly, the BBAT score is a four‐part score completed by healthcare professionals [18]. Components include evaluation of mother and baby positioning, success of attachment, sucking patterns and effective milk transfer. The BBAT provides a simple, rapid and accurate method of breastfeeding session appraisal to guide healthcare providers in their advice to support mothers to continue breastfeeding [18]. The LATCH score is a comparable, 10‐point breastfeeding assessment score, with five components assessing Latch, Audible swallowing, Type of nipple, Comfort and Help from the mother to support the infant at the breast, scored 0–2 depending on component success [19]. The MVCBAS is a similar tool assessing the mothers’ experience of breastfeeding, relating to maternal perception of factors such as positioning, latch and suckling at the breast [20]. Each tool is validated for assessment of breastfeeding outcomes in the postpartum period [21].

**TABLE 1:** Study characteristics for the included studies in our analysis.

Study	Country	Nipple grade	Age group	Stage of pregnancy	Hoffman’s exercise technique	Control group technique	Assessment time	Measurement instrument	Risk of bias
Padmavathi, 2015	India	Flat and retracted nipples	21–25 years	Immediate post‐partum	5 times per day for 3 days; thumb or forefinger pressed into the breast tissue and pushed away from the areola	Routine care	4th day postpartum	MVCBAS	Moderate concerns
Bulbuli et al., 2018	India	Flat and inverted nipples	18–30 years	Immediate post‐partum	5 times per day for 4 days; physiotherapy followed by Hoffman’s exercise (thumb and forefinger placed at the nipple base, pressed into tissue, then pulled apart. Complete 5 repetitions in the horizontal plane and 5 repetitions in the vertical plane)	Routine care with conventional physiotherapy using hot moist pack (43–46°C) for 10 mins, twice daily for 4 days and manual massage (kneading from areola) for ∼15 mins	4th day postpartum	LATCH	Some concerns
Kaur et al., 2020	India	Flat and inverted nipples	20–35 years	From 37 to 40 gestational weeks until delivery	5 times per day until delivery; stretching nipple horizontally and vertically with thumbs and forefinger	Routine care	Immediately after delivery	MVCBAS	Serious concerns
Thurkkada et al., 2022	India	Grade I inverted nipples	18–38 years	2–4 h postpartum	4 times per day (30 min each) for; thumbs placed at the nipple base, pressed and pulled horizontally and vertically	Routine care	3rd day postpartum	BBAT	Low concerns
Ahmed et al., 2024	Egypt	Grade I inverted nipples	20–35 years	From 36 gestational weeks until delivery	3–4 times per day (30 min each) for 6 weeks; thumbs placed at the nipple base, pulled apart horizontally and vertically, rotated around base	Inverted syringe technique: 3–4 times per day (5 min each) for 6 weeks and before each feeding session after delivery; using a 20 mL syringe modified to apply suction	6th week after the start of interventions	MVCBAS	Serious concerns
Belal et al., 2024	Egypt	Grade I or II inverted nipples	18–32 years	From 37 to 42 gestational weeks until delivery	5 times per day; thumbs placed at the base of nipple, pressed into the tissue, then pulled apart horizontally and vertically	Inverted syringe technique: 10 mL syringe modified to create suction; used several times per day before feeding	End of postpartum period	LATCH	Serious concerns
Elkhatib et al., 2024	Egypt	Flat or inverted nipples	18–24 years	Immediate post‐partum	5 times per day (10 min each) for 2 weeks; thumbs placed at the nipple base, pulled apart horizontally and vertically	Routine care	15th day postpartum	LATCH	Serious concerns
Preethi et al., 2024	India	Flat or inverted nipples	At least 21 years or older	Immediate post‐partum	5 times per day (3–5 min each) for 4 days; thumbs placed opposite at the base, pulled apart and rolled	No control group	4th day postpartum	BBAT	Serious concerns
MR and Jose, 2024	India	Flat or inverted nipples	At least 18 years or older	Immediate post‐partum	5 times per day for 3 days; thumbs placed opposite at the base, pulled apart up, down and sideways	No control group	3rd day postpartum	MVCBAS	Moderate concerns
Nikita et al., 2025	India	Flat and retracted nipples	At least 20 years or older	Immediate post‐partum	4 times per day for 2 days; thumbs placed opposite at the base, pulled apart gently and firmly	Inverted syringe technique: 4 times per day for 2 days; 10 mL syringe cut and used to create suction on nipple	2nd day postpartum	MVCBAS	Critical concerns

*Note:* LATCH: latch, audible swallowing, type of nipple, breast/nipple comfort and hold positioning.

Abbreviations: BBAT, Bristol Breastfeeding Assessment Tool; MVCBAS, Modified Via Christi Breastfeeding Assessment Scale.

The data were extracted using a standardised Microsoft Excel template sheet by NJ and verified by HL and ICCK. The following data were extracted—(a) study characteristics such as authors, year of publication, study design, sample size and country of investigation; (b) patient demographics such as age, stage of pregnancy, nipple inversion grade; (c) intervention specifics such as technique, duration, frequency in both control and Hoffman’s exercise group; and (d) assessment instrument specifics including questionnaire name and administration time point. None of the included studies reported any funding or financial support. Quality appraisal was done using pre‐defined tools—Cochrane Risk of Bias tool (ROB2) for randomised controlled trials and ROBINS‐I for interventional studies. These tools have several domains that are rated as low, high, or unclear risk of bias.

Our quality appraisal rated the randomised controlled trials as having low to some concerns; however, most of the interventional studies were rated as having high to critical risk of bias (Figure 1; Table 1) [22, 23]. These findings mirror prior reviews [4]. Given this anticipated limitation, and the small evidence base (*n* = 10 studies), a conventional, confirmatory meta‐analysis would have been inappropriate and potentially misleading. In keeping with this stance, we avoided strong certainty language (e.g., “effective” or “proven”) and did not grade the body of evidence beyond noting its very low certainty due to bias, inconsistency, and imprecision. For the exploratory meta‐analysis, we extracted the total number of patients in each group, the mean total score on the questionnaire and standard deviation. To support interpretation under uncertainty, we also report 95% confidence interval (95% CI) and prediction interval (PI) for the pooled estimates. The purpose of the next section was hence only exploratory in nature vis‐à‐vis to surface methodological shortcomings, provide transparent quantitative context to current claims and set out a research roadmap. All analyses were done in R v4.4.1 using R studio. The Preferred Reporting Items for Systematic reviews and Meta‐Analyses extension for Scoping Reviews (PRISMA‐ScR) Checklist is provided in Supporting File 1.

## 4. Results—Primary Outcome

We relied on an inverse‐variance random‐effects model of between‐group differences using Hedges’ g standardised mean difference (SMD). The pooled effect supported the general notion that Hoffman’s exercise may improve breastfeeding uptake in comparison to control groups (overall pooled *g* = 1.28; 95% CI: 0.12–2.44; PI: −2.14–4.7). The between‐study heterogeneity was high as seen by τ^2^ = 1.84; *I* ^2^ = 96% (Figure 2(a)).

FIGURE 2Forest plot and funnel plots for between‐group standardised mean differences (Hedges’ g) for Hoffman exercises vs. control, sub‐grouped by comparator type. (a) Positive values favour the experiment group. Hedges’ g was calculated with a small‐sample bias correction. Squares represent study estimates with area proportional to the inverse‐variance weight; horizontal black lines are 95% confidence intervals (95% CIs). Blue diamond shows random‐effects pooled estimates (REML estimator with Hartung–Knapp adjustments). The horizontal red bars are the corresponding prediction intervals (PIs). (b) Baujat plot showing the contribution of each study to the pooled effect (*y*‐axis) and to heterogeneity Q (*x*‐axis) (c) Contour‐enhanced funnel plot and (d) trim‐and‐fill funnel plot with three imputed studies (yellow dots) for the overall model. Shaded regions denote two‐sided *p* < 0.10, < 0.05, and < 0.01. Grey dots represent individual study estimates.

**Figure:**
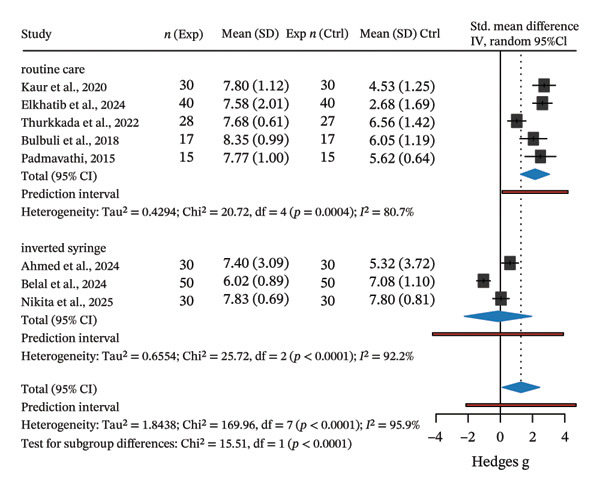
(a)

**Figure:**
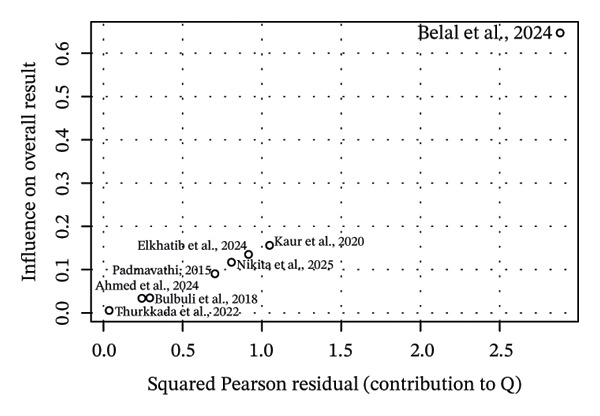
(b)

**Figure:**
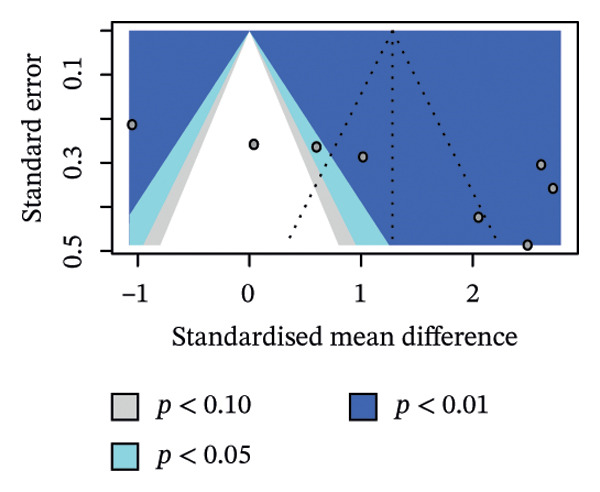
(c)

**Figure:**
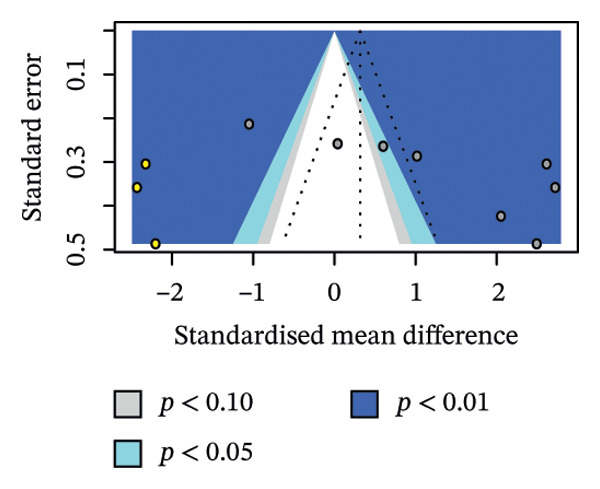
(d)

A meta‐regression analysis showed that the bulk of the heterogeneity originated from the differences in control comparator (*R* ^2^ = 72.2%) rather than the assessment instrument used (*R* ^2^ = 0%). Subsetting our dataset based on the control comparator further demonstrated that compared to no intervention (i.e., routine care), Hoffman’s exercise may be efficacious in increasing breastfeeding uptake (*g* = 2.16; 95% CI: 1.26–3.05; PI: 0.11–4.2). At the same time, we found no differences in efficacy between Hoffman’s exercise and inverted syringe technique (*g* = −0.15; 95% CI: −2.24–1.95; PI: −4.22–3.92).

Influence sensitivity diagnostics using studentised deleted residuals, Cook’s distance, and Baujat plot identified one influential study (Figure 2(b)). Excluding this study [13] increased the overall pooled effect from *g* = 1.28 to *g* = 1.62 and reduced between‐study heterogeneity from *τ* ^2^ = 1.84 to *τ* ^2^ = 1.09. This indicated that the study by Belal et al. [13] was pulling the estimate toward the null and inflating *τ* ^2^. We also looked at potential publication bias in the overall estimate as an exploratory analysis. The contour‐enhanced funnel plot showed scattering consistent with high between‐study heterogeneity (Figure 2(c)).

Egger’s test, with a relaxed *k* threshold, suggested asymmetry (intercept 15.79, *p* = 0.014). To adjust for this bias and small study effects, we ran a limit meta‐analysis, which shifted the overall pooled estimate from *g* = 1.28 to *g* = −0.45 (95% CI: −2.51–1.60), offsetting the mild positive effect in favour of Hoffman’s exercise we observed in unadjusted analysis. Trim‐and‐fill imputed three hypothetical studies and moved the estimate to *g* = 0.32 (95% CI: −1.06–1.69; Figure 2(d)). Of note, these estimates are not fully reliable due to *k* < 10, and we therefore treat them as sensitivity checks only; our main interpretation remains unchanged.

## 5. Results—Secondary Outcome

We next performed a random‐effects meta‐analyses of within‐exercise group pre–post changes using SMD with an assumption of pre–post correlation *r* = 0.5 (Figure 3). The pooled effect supported the notion that Hoffman’s exercise can improve breastfeeding (*g* = 3.95; 95% CI: 2.70–5.19; PI: 0.73–7.16). The between‐study heterogeneity was high as seen by τ^2^ = 1.47; *I* ^2^ ≈ 86%, indicating considerable dispersion of effects across studies.

FIGURE 3Forest plot of within‐group pre–post meta‐analysis, sub‐grouped by assessment instrument (a) and sensitivity plot to the assumed pre–post correlation (b). (a) Study‐level effects are shown using standardised mean changes (Hedges’ g; SMCR) treating observations as paired and using an assumed pre–post correlation *r* = 0.5 to obtain sampling variances. Positive values favour the experiment group. Squares represent study estimates with area proportional to the inverse‐variance weight; horizontal black lines are 95% confidence intervals (95% CIs). Diamonds show random‐effects pooled estimates (REML estimator with Hartung–Knapp adjustments). The horizontal red bars are the corresponding prediction intervals (PIs). (b) Sensitivity of the analysis to the assumed pre–post correlation (*r* = 0.1–0.9). For each r, SMCR variances were recomputed and the overall random‐effects model re‐fit (REML with Hartung–Knapp adjustments). The black line shows *τ* ^2^ (left *y*‐axis). The blue points/lines show the pooled Hedges’ g with 95% CI, plotted against r and displayed on a secondary *y*‐axis (linear rescaling).

**Figure:**
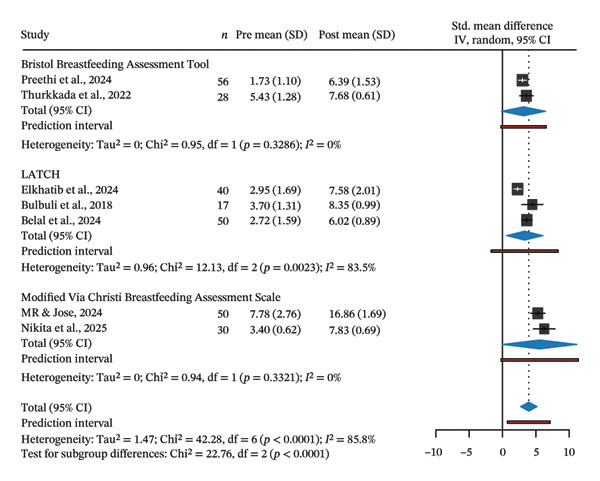
(a)

**Figure:**
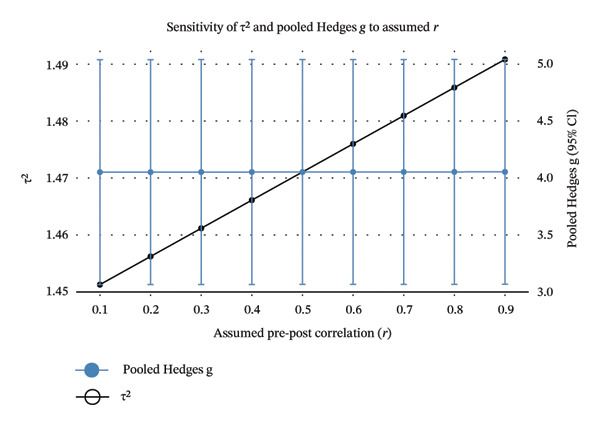
(b)

When stratified by assessment instrument, the pooled effects were BBAT *g* = 3.16 (95% CI: 0.17–6.48; PI: 0.24–6.55), LATCH *g* = 3.32 (95% CI: 0.59–6.06; PI: 1.71–8.35) and MVCBAS *g* = 5.58 (95% CI: 0.04–11.20; PI: 0.22–11.40). These findings confirmed that while all instruments captured large average improvements from baseline, precision was limited for BBAT and MVCBAS. At the same time, heterogeneity was substantial for LATCH. Sensitivity analysis by varying the assumed pre–post correlation from *r* = 0.10 to 0.90 showed that the pooled mean essentially remains unchanged (*g* ≈ 3.9), as expected since SMD estimates do not depend on *r*. However, model heterogeneity increased slightly with larger *r* (*τ* ^2^ ≈ 1.45 to 1.49; *I* ^2^ ≈ 86% to 89%), indicating that our conclusions are robust to reasonable *r* while the dispersion of true effects remained wide.

## 6. Mechanism of Action

Hoffman’s exercise is a nonsurgical method of inverted nipple repair, involving application of circum‐areolar downward traction to the nipple–areolar complex in a repetitive, regular fashion, with the aim to produce gradual nipple eversion over time. Several physiological mechanisms are postulated that underpin the observed clinical effects. The leading theory is that repetitive mechanical stretch produces remodelling of the dermal collagen and elastin fibre network. This affects the collagen turnover through mechano‐transduction and altered extracellular matrix production in fibroblasts, leading to reorientation of collagen fibres along stress lines to modify the nipple tissue compliance over time [24].

Disruption of subareolar fibrous bands with lengthening of connective tissue through mechanical creep and stress relaxation simultaneously produces tissue expansion in line with understood mechanisms associated with other techniques using implant‐based expanders [25]. Specific to the nipple–areolar complex, mechanical stretch and disruption of adhesion can produce reorientation of ductal structures through improved tissue compliance, improving duct mobility [26]. Repetitive manual manipulation may moreover influence smooth muscle through reduction of hypertonicity and reflect contraction in the smooth muscle surrounding the nipple shaft, reducing resistance or eversion. Mechanical stimulation is well understood to promote vasodilation and improved capillary perfusion, extracellular fluid turnover, and improved tissue viability.

Neural and somatosensory effectives of repetitive manipulation of the nipple–areolar complex reduces sympathetic tone and local inhibitory reflexes. These mechanisms remain largely theoretical without high‐quality imaged‐based mechanistic data to support the claims.Nonetheless, the physiology of tissue expansion and collagen remodelling in management of scarring has been well described [27]. We believe it could provide a conceptual evidence base for Hoffman’s exercise effectiveness. Previous research has described microscopic findings of mechanical stretch on the skin in mice *ex vivo*. Under traction, collagen fibres were found to dynamically reorient and align along the direction of stretch, suggesting that traction can reorganise collagen microarchitecture in dermal tissues [28]. This is further supported by Davis’s law, which describes how soft tissue models along imposed external demands. Repetitive circum‐areolar traction produces elongation of soft tissue fibres through the remodelling of collagen, elastin and smooth muscle and addition of new cellular matrices [29]. Application of advanced imaging techniques (e.g., ultrasound, Doppler, 3D imaging) in future studies would help validate the proposed mechanisms.

## 7. Clinical Interpretation of the Findings

Our findings indicate that when studies were grouped by comparator, pooled effects suggested greater breastfeeding success with Hoffman’s exercise than with routine care. However, this directionality did not extend to comparisons with the inverted syringe, where effects were small and highly uncertain. Given the high risk of bias in several studies, variable and often undescribed ‘*routine care*’ protocols and wide prediction intervals, we judge the comparative effectiveness as uncertain, with any apparent benefit driven largely by contrasts against minimal intervention. Similarly, pre–post standardized mean changes within Hoffman cohorts (non‐causal) were large, indicating short‐term improvement on scale scores. Because the estimates are uncontrolled for confounders and came from small cohort sizes, they should be viewed as hypothesis‐generating rather than confirmatory.

Controlled designs with baseline‐adjusted analyses (e.g., ANCOVA) are needed to determine the incremental benefit of Hoffman’s exercise over specific comparators. Differences between BBAT, MVCBAS and LATCH scoring scales likely reflect not only structural variability but also differences in outcome assessment timings and cohort‐mix. BBAT and MVCBAS demonstrated internal consistency across studies, while LATCH appeared more responsive to short‐term change. Because instruments were not applied uniformly and anchor‐based minimal important differences (MCIDs) are lacking, we caution against instrument‐to‐instrument conclusions. A core outcome set and harmonised assessment time points are needed, alongside validation work on responsiveness and clinically important change.

Taken together, we do not suggest a complete abandoning of Hoffman’s exercise. At the same time, we believe the evidence does not justify for the exercise to be presented as proven, effective, or as a superior therapy, at least not as a stand‐alone intervention. We recommend that in routine care, clinicians should embed nipple eversion strategies within a broader protocolised breastfeeding‐support bundle that includes skin‐to‐skin contact, frequent coached attempts, deep‐latch positioning, hand expression, early lactation‐consultant input and prompt treatment of nipple trauma [30]. Hoffman’s exercise may be offered as an optional, low‐risk adjunct with clear instructions.

The patients must be instructed on the technique for applying the traction forces in a time‐limited trial (e.g., 3–7 days). Teaching should emphasise gentle technique (no excessive force; no prolonged ischaemic pressure) and defer manipulation on acutely traumatised or infected nipples until healed. Clinicians should be prepared to escalate or switch if comfort or latch does not improve promptly. We highly recommend collecting baseline measurements to assess clinical and subjective progress. Regarding the choice of assessment tool, there is no evidence supporting one over the other—the final choice rests with the clinician. We, regardless of the tool, do recommend monitoring for key outcomes including exclusive/any breastfeeding, maternal pain/trauma, need to switch technique and adverse effects.

Ultimately, whether to administer Hoffman’s exercise or not should be individualised through shared decision‐making, acknowledging that evidence for benefit is uncertain. For higher‐grade inversion or where rapid eversion is needed to establish latch, clinicians may preferentially use an active device during feeds while maintaining identical co‐interventions across approaches. Framed in this way, as a short‐term, supervised adjunct within a standardised support package, the use of Hoffman’s exercise remains consistent with the current, low‐certainty evidence while avoiding overstatement of its comparative effectiveness.

## 8. Recommendations for Future Studies

Based on our synthesis, we suggest that future studies and trial should

a. Run head‐to‐head superiority trials comparing with routine care. It would be essential to clearly define routine care a priori (e.g., educational only or with physiotherapy; counselling content, latch coaching, frequency, provider).
b. Run head‐to‐head non‐inferiority trials with the inverted‐syringe technique. This would include pre‐specification and justification of the non‐inferiority margin, along with keeping co‐interventions identical. Appropriate double‐blinding measures must be implemented.
c. Compare and validate breastfeeding assessment tools (BBAT, MVCBAS, LATCH) by assessing responsiveness, test–retest, inter‐rater reliability and anchor‐based minimal important change in this population.
d. Diversify settings and populations beyond two countries (India and Egypt). Multicentre studies across varied health systems (urban/rural; low income/high income countries) should be undertaken considering the differences in staffing and lactation‐consultant availability.
e. Compare the effectiveness by the stage the condition, i.e., Grade I vs II. Consider stratified randomisation or pre‐specified subgroup analyses to achieve necessary statistical power and sensitivity.
f. Stratify and compare effectiveness by maternal characteristics. This can be done by pre‐specifying age bands (e.g., 18–25, 26–35, ≥ 36), parity (primipara vs multipara), mode of delivery, BMI/diabetes and nipple trauma at baseline. It is important to also report the cause of nipple defect (congenital vs acquired).
g. Compare different time points for initiating the exercise, i.e., compare antenatal vs postpartum initiation and early (first 24–48 h) vs later start. Examine dose–timing interactions.
h. Optimise the exercise technique and intensity. Factorial or multi‐arm trials are needed to identify frequency, session length and overall duration of exercise that balances benefit and burden.
i. Define validated endpoints for “success/failure” and escalation criteria (e.g., switch to device if no latch improvement by 48–72 h or if pain increases beyond a certain threshold).
j. Consider reporting secondary patient‐reported outcomes such as exclusive/any breastfeeding at key time points, maternal pain/trauma, infections/mastitis, discontinuation due to discomfort, satisfaction/acceptability.
k. Minimise fidelity and document patient adherence. It is necessary to document who teaches, how (in‐person or online), skills check for the patient, number of sessions completed and reasons for non‐adherence.

Higher‐quality evidence for the effectiveness of Hoffman’s exercises in improving nipple inversion and breastfeeding will facilitate appropriate, and reliable comparisons to other non‐surgical interventions including a range of gradual traction methods [31]. Understanding the definitive evidence relating to nonsurgical methods of treatment for Grade I and II inverted nipples will allow practitioners to be well informed in their clinical practice and patient counselling, to improve both aesthetic and functional outcomes in this cohort [32].

## 9. Recommended Sample Size for Future Studies

To support future studies, we here present briefly sample size estimates from our pooled analyses. In our between‐group analysis, because effect size in Hoffman’s exercise versus routine care were large but the PI lower bound was modest, we recommend considering an expected effect size in the range of *d* = 0.4—0.5. For superiority analysis with unadjusted two‐samples *t*‐test, about 63 participants per arm would be needed, while about 50 participants per arm for ANCOVA (assuming *ρ* ≈ 0.5; 1:1 allocation) would be needed. Conversely, when comparing Hoffman’s exercise with an inverted syringe, we observed no clear advantage, so superiority trials would need to be designed to detect a small effect (*d* = 0.2–0.3), implying much larger samples (about 300–400 participants per arm with ANCOVA at *ρ* = 0.5, *α* = 0.05, 80% power). We rather recommend considering a non‐inferiority design with a justified margin that would require smaller sample sizes per trial arm.

For planning future single‐group pre–post studies, we suggest targeting a conservative SMD equal to the PI lower bound from the meta‐analysis (*g* = 0.73). This corresponds to about 18 participants for 80% power using a non‐parametric Wilcoxon signed‐rank test at α = 0.05. For two‐arm pre–post trials, as prior controlled evidence is unavailable, a cautious target of *d* = 0.4–0.5 would be ideal. This would imply ≥ 50 participants per arm for 80% power using ANCOVA at α = 0.05 with no covariates considered (1:1 allocation).

## 10. Conclusions

Our exploratory findings suggest that any apparent advantage of Hoffman’s exercise is largely driven by contrasts with minimal or poorly described care, while head‐to‐head comparisons with routine care and active techniques (such as inverted syringe) remain under‐standardised and imprecise. Until future trials reporting protocolised comparators and standardised interventions are conducted, the comparative effectiveness should be considered clinically uncertain. Finally, as a reminder, our pooled estimates must be viewed as hypothesis‐generating rather than definitive.

## Author Contributions

Nityanand Jain and Hester Lacey conceptualised the study and were responsible for methodology. Data analyses and visualisations were done by Nityanand Jain. Data collection and curation were done by Nityanand Jain, Hester Lacey and Ian C. C. King. Project supervision, investigations and resource management were led by Ian C. C. King. Original draft was prepared by Nityanand Jain and Hester Lacey, while revisions and final editing were done by all authors.

## Funding

Open access publishing facilitated by Flinders University, as part of the Wiley ‐ Flinders University agreement via the Council of Australian University Librarians.

## Disclosure

All authors have read and approved the final manuscript for publication. The views and findings expressed in the article are those of the authors and do not necessarily reflect those of the affiliated institutions or the publisher.

## Ethics Statement

The authors have nothing to report since all data were sourced from already published materials in the literature.

## Consent

The authors have nothing to report.

## Conflicts of Interest

The authors declare no conflicts of interest.

## Supporting Information

Supporting File 1. Preferred Reporting Items for Systematic reviews and Meta‐Analyses extension for Scoping Reviews (PRISMA‐ScR) Checklist.

## Supporting information


**Supporting Information** Additional supporting information can be found online in the Supporting Information section.

## Data Availability

All data analysed in the present study were gathered from open‐access resources, which are freely available in the public domain. R code, extraction sheets and other data generated in the analysis can be obtained from the corresponding author on reasonable request.

## References

[1] Nagaraja Rao D. and Winters R. , Inverted Nipple, 2025, StatPearls Publishing, https://www.ncbi.nlm.nih.gov/books/NBK563190/. 33085337

[2] Park H. S. , Yoon C. H. , and Kim H. J. , The Prevalence of Congenital Inverted Nipple, Aesthetic Plastic Surgery. (March 1999) 23, no. 2, 144–146, 10.1007/s002669900258, 2-s2.0-0344780828. 10227917

[3] Han S. and Hong Y. G. , The Inverted Nipple: Its Grading and Surgical Correction, Plastic and Reconstructive Surgery. (August 1999) 104, no. 2, 389–395, 10.1097/00006534-199908000-00010, 2-s2.0-0344780714. 10654681

[4] Kaya Ö. , Tecik S. , Suzan ÖK. , Kabul F. , Koyuncu O. , and Çınar N. , The Effect of Interventions on Flat and Inverted Nipple on Breastfeeding: A Systematic Review, Journal of Pediatric Nursing. (January 2024) 74, e1–e13, 10.1016/j.pedn.2023.07.024. 37558567

[5] Abd-Ella N. Y. A. and Mohammed S. F. , Effectiveness of Hoffman’s Exercise on the Level of Breastfeeding Among Primiparous Women With Inverted Nipple, Egyptian Journal of Health Care. (2021) 12, no. 1, 607–624, https://ejhc.journals.ekb.eg/article_143538_a3cf76e3ad14bbf09371b7d6da1ece66.pdf, 10.21608/ejhc.2021.143538.

[6] Ghosh D. and Singh A. , Effectiveness of Hoffman Exercise on Breastfeeding Among Primipara Mothers With Flat and Retracted Nipple: A Narrative Review, International Journal of Nursing Education. (2019) 11, no. 4, 44–45, 10.37506/ijone.v11i4.3948.

[7] Thurkkada A. P. , Rajasekharan Nair S. , Thomas S. et al., Effectiveness of Hoffman’s Exercise in Postnatal Mothers With Grade 1 Inverted Nipples, Journal of Human Lactation. (February 2023) 39, no. 1, 69–75, 10.1177/08903344221102890. 35695389

[8] Safitriana S. , Budiati T. , and Rachmawati I. N. , Management of Breast and Nipple Problems in Breastfeeding Mothers: Systematic Review. Disease Prevention and Public, Health Journal. (2024) 18, no. 1, 47–61, 10.12928/dpphj.v18i1.10274.

[9] Mr R. and Jose S. , Effect of Hoffman’s Exercise on Level of Breastfeeding Among Postnatal Mothers With Nipple Defect, International Journal of Obstetrics and Gynaecological Nursing. (2024) 6, no. 1, 163–166, 10.33545/26642298.2024.v6.i1c.149.

[10] Preethi B. , Annal M. A. , Lavanya S. , Poongodi V. , and Umamaheswari R. , Effectiveness of Hoffman’s Exercise on Breastfeeding Among Postnatal Mothers With Flat or Inverted Nipple in Tertiary Care Hospital, Puducherry, Chettinad Health City Medical Journal. (2024) 13, no. 1, 56–60, 10.24321/2278.2044.202410.

[11] Nikita R. , Prajapati A. , and Patel K. , Assess Effectiveness of Hoffman’s Exercise V/S Syringe Technique on Level of Successful Breast Feeding Among Primipara Mothers With Flat and Retracted Nipples Admitted in Svbch, Silvassa, International Journal of Multidisciplinary Research. (2025) 7, no. 2, 1–12, 10.36948/ijfmr.2025.v07i02.39129.

[12] Kaur A. , Saini P. , and Sharma K. , A Study to Evaluate the Effectiveness of Hoffman’s Exercise on Successful Breastfeeding Among Antenatal Mothers With Nipple Defects at Sri Guru Ram Das Hospital, Vallah, Amritsar, Punjab, International Journal of Health Sciences and Research. (2020) 10, no. 3, 121–128, https://www.ijhsr.org/IJHSR_Vol.10_Issue.3_March2020/19.pdf.

[13] Belal G. A. E. S. , Gomaa M. M. , and Abdelmenem E. E. , Effect of Nursing Interventions Strategies for Inverted Nipple on Efficiency of Early Breastfeeding Among Primiparous Mothers, Assiut Scientific Nursing Journal. (2024) 12, no. 45, 341–358, 10.21608/asnj.2024.306764.1868.

[14] Padmavathi P. , Effectiveness of Hoffman’s Exercise on Successful Breast Feeding Among Primipara Mothers With Flat and Retracted Nipples, International Journal of Nursing Education and Research. (2015) 3, no. 2, 124–126, https://ijneronline.com/AbstractView.aspx?PID=2015-3-2-6https://ijneronline.com/AbstractView.aspx?PID=2015-3-2-6.

[15] Bulbuli A. , Fernandes S. , and Shelke S. , Effect of Hoffman’s Exercises on Flat or Inverted Nipples in Immediate Postpartum Mothers–A Randomized Control Trial, Indian Journal of Physiotherapy & Occupational Therapy. (2018) 12, no. 3, 88–92, 10.5958/0973-5674.2018.00063.1.

[16] Elkhatib H. M. A. , Kandeel H. A. E. M. , and Elgmal E. G. R. , Effect of Hoffman’s Exercise on Successful Breastfeeding and Satisfaction Among Postnatal Women With Flat and Inverted Nipples, Egyptian Journal of Health Care. (2024) 15, no. 2, 89–101, 10.21608/ejhc.2024.350726.

[17] Ahmed A. H. , Taman A. H. S. , and Hassan S. I. , Effect of Hoffman’s Exercise Versus Inverted Syringe Technique on Nipple Length and Breastfeeding Outcomes Among Primiparous Women With Inverted Nipple, Egyptian Journal of Health Care. (2024) 15, no. 3, 1972–1991, 10.21608/ejhc.2025.443793.

[18] Ingram J. , Johnson D. , Copeland M. , Churchill C. , and Taylor H. , The Development of a New Breast Feeding Assessment Tool and the Relationship With Breast Feeding Self-Efficacy, Midwifery. (January 2015) 31, no. 1, 132–137, 10.1016/j.midw.2014.07.001, 2-s2.0-84920599755. 25061006 PMC4275601

[19] Jensen D. , Wallace S. , and Kelsay P. , LATCH: A Breastfeeding Charting System and Documentation Tool, Journal of Obstetric, Gynecologic, and Neonatal Nursing. (January 1994) 23, no. 1, 27–32, 10.1111/j.1552-6909.1994.tb01847. 8176525

[20] Brugaletta C. , Le Roch K. , Saxton J. , Bizouerne C. , McGrath M. , and Kerac M. , Breastfeeding Assessment Tools for at-Risk and Malnourished Infants Aged Under 6 Months Old: A Systematic Review, F1000Res. (November 2020) 9, 10.12688/f1000research.24516.2. PMC7898355 33628437

[21] Chapman D. J. , Doughty K. , Mullin E. M. , and Pérez-Escamilla R. , Reliability of Lactation Assessment Tools Applied to Overweight and Obese Women, Journal of Human Lactation. (May 2016) 32, no. 2, 269–276, 10.1177/0890334415597903, 2-s2.0-84963985303. 26243754

[22] Sterne J. A. C. , Savović J. , Page M. J. et al., RoB 2: A Revised Tool for Assessing Risk of Bias in Randomised Trials, BMJ. (August 2019) 366, 10.1136/bmj.l4898, 2-s2.0-85071628750. 31462531

[23] Sterne J. A. , Hernán M. A. , Reeves B. C. et al., ROBINS-I: A Tool for Assessing Risk of Bias in Non-Randomised Studies of Interventions, BMJ. (October 2016) 355, 10.1136/bmj.i4919, 2-s2.0-84991710934. PMC5062054 27733354

[24] Hong Y. , Peng X. , Yu H. et al., Cell-Matrix Feedback Controls Stretch-Induced Cellular Memory and Fibroblast Activation, Proceedings of the National Academy of Sciences of the United States of America. (March 2025) 122, no. 12, 10.1073/pnas.2322762122. PMC11962495 40100625

[25] Yang F. , Das D. , and Chasiotis I. , Microscale Creep and Stress Relaxation Experiments With Individual Collagen Fibrils, Optics and Lasers in Engineering. (March 2022) 150, 10.1016/j.optlaseng.2021.106869. PMC8752082 35027783

[26] Mangialardi M. L. , Baldelli I. , Salgarello M. , and Raposio E. , Surgical Correction of Inverted Nipples, Plast Reconstr Surg Glob Open. (July 2020) 8, no. 7, 10.1097/GOX.0000000000002971. PMC7413770 32802664

[27] Xu F. , Lu T. J. , and Seffen K. A. , Biothermomechanical Behavior of Skin Tissue, Acta Mechanica Sinica. (2008) 24, 1–23, 10.1007/s10409-007-0128-8, 2-s2.0-37749041501.

[28] Ducourthial G. , Affagard J. S. , Schmeltz M. et al., Monitoring Dynamic Collagen Reorganization During Skin Stretching With Fast Polarization-Resolved Second Harmonic Generation Imaging, Journal of Biophotonics. (May 2019) 12, no. 5, 10.1002/jbio.201800336, 2-s2.0-85060678058. 30604478

[29] Nutt J. J. , Diseases and Deformities of the Foot, Davis’s Law. E.B. Treat and Company, NY, USA. (1913) 1–313, https://archive.org/details/diseasesanddefo00nuttgoog/page/n208/mode/2up.

[30] Coca K. P. , Pinto V. L. , Westphal F. , Mania P. N. A. , and Abrão A. C. F. V. , Bundle of Measures to Support Intrahospital Exclusive Breastfeeding: Evidence of Systematic Reviews, Revista Paulista de Pediatria. (April 2018) 36, no. 2, 214–220, 10.1590/1984-0462/. 29694490 PMC6038790

[31] Ritz M. , Silfen R. , Morgan D. , and Southwick G. , Simple Technique for Inverted Nipple Correction, Aesthetic Plastic Surgery. (January 2005) 29, no. 1, 24–27, 10.1007/s00266-004-0044-2, 2-s2.0-18844434686. 15583847

[32] Olivas-Menayo J. and Berniz C. , Inverted Nipple Correction Techniques: An Algorithm Based on Scientific Evidence, Patients’ Expectations and Potential Complications, Aesthetic Plastic Surgery. (April 2021) 45, no. 2, 472–480, 10.1007/s00266-020-01909-6. 32754835

